# Muscle Wasting and Resistance of Muscle Anabolism: The “Anabolic Threshold Concept” for Adapted Nutritional Strategies during Sarcopenia

**DOI:** 10.1100/2012/269531

**Published:** 2012-12-23

**Authors:** Dominique Dardevet, Didier Rémond, Marie-Agnès Peyron, Isabelle Papet, Isabelle Savary-Auzeloux, Laurent Mosoni

**Affiliations:** ^1^Clermont Université and Unité de Nutrition Humaine, Université d'Auvergne, BP 10448, 63000 Clermont-Ferrand, France; ^2^INRA, UMR 1019, UNH, CRNH Auvergne, 63000 Clermont-Ferrand, France; ^3^UNH Centre de Clermont-Ferrand-Theix, INRA, 63122 Saint Genès, France

## Abstract

Skeletal muscle loss is observed in several physiopathological situations. Strategies to prevent, slow down, or increase recovery of muscle have already been tested. Besides exercise, nutrition, and more particularly protein nutrition based on increased amino acid, leucine or the quality of protein intake has generated positive acute postprandial effect on muscle protein anabolism. However, on the long term, these nutritional strategies have often failed in improving muscle mass even if given for long periods of time in both humans and rodent models. Muscle mass loss situations have been often correlated to a resistance of muscle protein anabolism to food intake which may be explained by an increase of the anabolic threshold toward the stimulatory effect of amino acids. In this paper, we will emphasize how this anabolic resistance may affect the intensity and the duration of the muscle anabolic response at the postprandial state and how it may explain the negative results obtained on the long term in the prevention of muscle mass. Sarcopenia, the muscle mass loss observed during aging, has been chosen to illustrate this concept but it may be kept in mind that it could be extended to any other catabolic states or recovery situations.

The main function of skeletal muscle is to provide power and strength for locomotion and posture, but this tissue is also the major reservoir of body proteins and amino acids. Thus, although the loss of muscle proteins has positive effects in the short term by providing amino acids to other tissues, an uncontrolled and sustained muscle wasting impairs movement, leads to difficulties in performing daily activities, and has detrimental metabolic consequences with reduced ability in mobilizing enough amino acids in case of illness and diseases. The resulting weakness increases the incidence of falls and the length of recovery and when advanced, muscle wasting is correlated to morbidity and increased mortality. Consequently, one of the challenges we have to face is to supply amino acids to the tissues with higher requirements in catabolic states [1] but also to prevent a too important loss in muscle proteins and ultimately improve muscle recovery. 

During the day, protein metabolism is modified by food intake. Whole-body proteins are stored during postprandial periods and lost in postabsorptive periods. With a muscle protein mass that remains constant, the loss of muscle proteins is compensated for the same protein gain in the postprandial state. In adult volunteers, oral feeding is associated with an increase in whole-body protein synthesis and a decrease in proteolysis [2–5]. These changes are mediated by feeding-induced increases in plasma concentrations of both nutrients and hormones. Many studies suggest that amino acids and insulin play major roles in promoting postprandial protein anabolism [6]. Thus, in case of muscle wasting, muscle protein loss results from an imbalance between protein accretion and break-down rates which, in part, comes from a defect in the postprandial anabolism. 

Although each muscle wasting situation is characterized by its specific mechanism(s) and pathways leading to muscle loss, an increase of catabolic factors such as glucocorticoids, cytokines, and oxidative stress, often occurs and it is now well established that these factors have potential deleterious effects on the amino acids or insulin signalling pathways involved in the stimulation of muscle anabolism after food intake [7–11].

These signalling alterations lead to an “anabolic resistance” of muscle even if the anabolic factor requirements (amino acids e.g.) are theoretically covered, that is, with a normal nutrient availability fitting the recommended dietary protein allowances in healthy subjects. This anabolic resistance may be in part explained by an increase of the muscle “anabolic threshold” required to promote maximal anabolism and protein retention (Figures 1(a) and 1(b)). Because the muscle “anabolic threshold” is higher, the anabolic stimuli (including aminoacidemia) cannot reach the anabolic threshold anymore and by consequence, muscle anabolism is reduced with the usual nutrient intake (Figure 1(b)). A possible nutritional strategy is then to increase the intake of anabolic factors (especially amino acids) to reach the new “anabolic threshold” (Figure 1(c)). There are several ways to increase amino acid availability to skeletal muscle: increase protein intake, to supplement the diet with one or several free amino acids or to select the protein source on its amino acid composition and physicochemical properties when digested in the digestive tract. These nutritional strategies tested to increase postprandial amino acid levels above the increased anabolic threshold and ultimately to restimulate muscle protein synthesis in situations of anabolic resistance led to conflicting results with no or more or less positive effects of the supplementation on nitrogen retention. This could be explained by variations in amino acid kinetics. The duration of the hyperaminoacidemia postprandially can also be of a variable magnitude and duration, depending of the form of the protein/amino acid supply in the diet. To illustrate this concept, we will take one example, that is, the loss of muscle mass during aging, while keeping in mind that this could be translated to any situation of muscle wasting.

Sarcopenia, as other catabolic states, has been found to result from a decreased response and/or sensitivity of protein synthesis and degradation to physiologic concentrations of amino acids [12–14]. This is related to a defect of the leucine signal to stimulate the mTOR signalling pathway activity [15]. These data suggest that increasing leucine availability may then represent a nutritional strategy to overcome the “anabolic threshold” increase observed during aging. Studies in both elderly humans and rodents subjected to free leucine supplementation have shown that such supplementations indeed acutely improved muscle protein balance after food intake by increasing muscle protein synthesis and decreasing muscle proteolysis in the postprandial state (reviewed in Balage and Dardevet [16]). However, the few chronic studies conducted with such free leucine supplementations did not succeed in promoting an increase in muscle mass [17–19]. Choosing free leucine as a supplement over a normal protein diet creates a desynchronization between leucine signal and the rise in all amino acids (Figure 2(a)). Indeed the free leucine is absorbed immediately whereas the other amino acids are released later after gastric emptying and proteolytic digestion in the gut. This nonsynchronization between the stimulation of muscle leucine-associated protein metabolism pathways and the delayed availability of amino acids as substrates can explain that protein anabolism was only stimulated for a very short period of time during the postprandial period and then could not translate into a significant muscle protein accretion. 

Studies with a synchronized leucine signal and amino acid availability have been performed by using leucine rich proteins that are rapidly digested (whey proteins) [20]. With such proteins, leucine availability is increased simultaneously with the other amino acids to reach the increased muscle anabolic threshold (Figure 2(b)). However, as observed for free leucine supplementation, when such dietary proteins where given on the long term in elderly rodents [21], muscle anabolism was acutely improved but muscle mass remained unchanged. However, Magne et al. [22] have shown that in elderly rodents recovering from acute muscle atrophy, leucine rich proteins were nevertheless efficient in improving recovery of muscle mass whereas free leucine supplementation remained ineffective. It may be postulated that, when given on the long term, protein muscle metabolism was adapted by increasing also protein catabolism in parallel with the increase of protein anabolism. However, after a catabolic state with an important muscle mass loss occurring within few days, this adaptation may be delayed and leucine rich proteins remained efficient in improving muscle mass.

According to these data, it can be concluded that besides counteracting the muscle anabolic resistance, the duration during which the anabolic resistance is muzzled also plays a critical role in leading to a significant muscle protein accretion. A prolonged stimulation could not be achieved with fast proteins at normal dietary level (even enriched with leucine) since the concentration of amino acids as substrates declines rapidly after their intake [23]. However, by strongly increasing protein intake, such ideal situations could be nevertheless achieved (Figure 2(c)). The “protein pulse feeding” initially developed by Arnal et al. [24–26] have shown that, by concentrating 80% of the total daily protein intake in one meal, protein retention was improved in elderly women subjected to a such nutritional strategy. Similarly, when very large amount of amino acids (wherein leucine formed the highest percentage of the mixture), positive results have been observed [27–30]. 

The above nutritional strategies discussed raised the problem that the organism has to cope with large amount of nitrogen to eliminate. This point can be critical with already frail sarcopenic subjects or patients for who the renal function will be oversolicited whereas it may be already altered. 

 In order to minimize this deleterious side effect of high protein intake, a strategy to reverse the increase in the “anabolic threshold” would restore the anabolic stimulation during the postprandial period with lower intake of dietary proteins or amino acid supplementations (Figures 3(a) and 3(b)). This requires the knowledge of the factors involved and responsible in the “anabolic threshold” elevation. The causes can be multiple and specific for each catabolic state. However, most of these muscle loss situations have in common an increase of the inflammatory status. Regarding aging, levels of inflammatory markers, such as interleukin-6 (IL6) and C reactive protein (CRP), increase slightly, and these higher levels are correlated with disability and mortality in humans [31, 32]. Even if the increase is moderate, higher levels of cytokines and CRP increase the risk of muscle strength loss [33] and are correlated with lower muscle mass in healthy older persons [34]. We have recently shown that the development of a low grade inflammation challenged negatively the anabolic effects of food intake on muscle protein metabolism and that the pharmacologic prevention of this inflammatory state was able to preserve muscle mass in old rodents [7, 8]. A resensitization of muscle protein synthesis to amino acids could be also achieved with other nutrients such as antioxidants [11, 35] but it is not known yet if such supplementations could be effective in preserving muscle mass. Interestingly, Smith et al. [36] have tested n-3 polyunsaturated fatty acids supplementation to increase the sensitivity of muscle protein metabolism to anabolic factors (amino acids and insulin) by increasing the cellular membrane fluidity in elderly volunteers. Although they obtained a resensitization of the mTOR signalling pathway with the n-3 fatty acids, it is not known if the decrease of the “anabolic threshold” has been large enough to translate into sufficient postprandial protein accretion and then preserve muscle mass in the long term if not associated with a concomitant dietary increase in amino acids. 

By choosing muscle mass loss during aging as an example of muscle wasting, it becomes obvious that skeletal muscle “anabolic threshold” is increased in such situations and that muscle protein metabolism becomes resistant to dietary anabolic factors even if these factors are supplied at the level they elicit maximal effects in normal physiological situations. It is important to note that this anabolic resistance during aging may be specific to amino acids [37]. Because the muscle “anabolic threshold” is more elevated, the duration of the stimulation by anabolic signals (as leucine) and the overcome of amino acid supply above the threshold is reduced with usual nutrient intake. Two strategies can be used (alone or in combination) to deal with this decreased “efficient” postprandial period: (1) by increasing the anabolic signals, and particular amino acid availability; however, it is necessary to synchronize the anabolic stimuli with the substrates in order to optimize the incorporation of amino acids into muscle proteins; (2) by increasing the efficiency of the postprandial period with strategies aiming at partially restoring (i.e., decreasing) the muscle “anabolic threshold”.

## Figures and Tables

**Figure 1:**
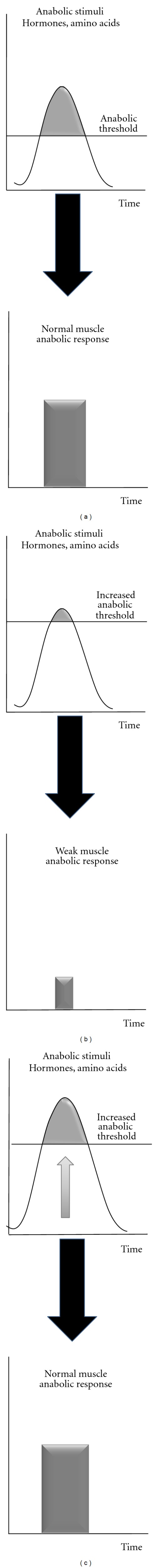
The concept of increased anabolic threshold with associated altered muscle protein anabolism during the postprandial period.

**Figure 2:**
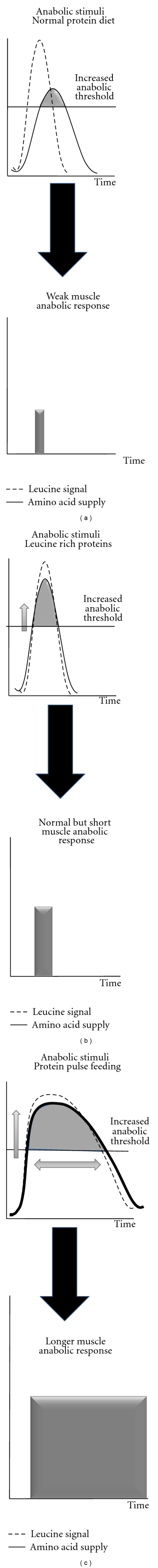
Free leucine, leucine rich proteins, and high protein diet in terms of amino acid kinetic and associated anabolic response in situation of increased muscle anabolic threshold.

**Figure 3:**
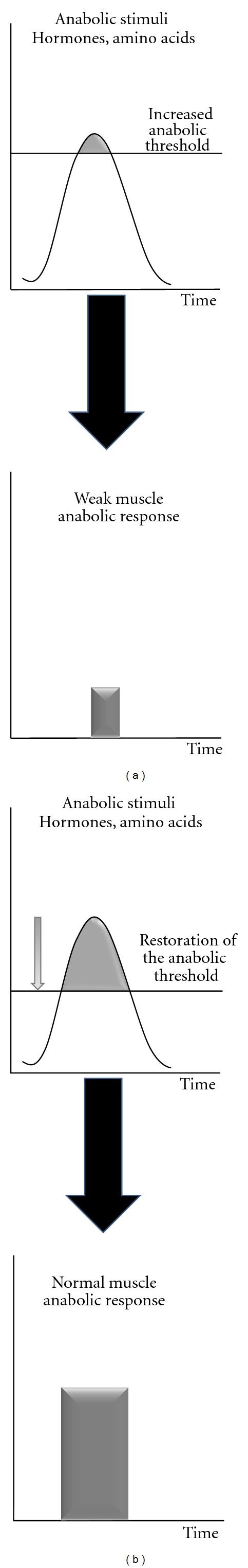
Strategies aiming at partially decreasing the muscle “anabolic threshold” and increasing the efficiency of the postprandial period.

## References

[1] Obled C, Papet I, Breuillé D (2002). Metabolic bases of amino acid requirements in acute diseases. *Current Opinion in Clinical Nutrition & Metabolic Care*.

[2] Rennie MJ, Edwards RHT, Halliday D (1982). Muscle protein synthesis measured by stable isotope techniques in man: the effects of feeding and fasting. *Clinical Science*.

[3] Pacy PJ, Price GM, Halliday D, Quevedo MR, Millward DJ (1994). Nitrogen homoeostasis in man: the diurnal responses of protein synthesis and degradation and amino acid oxidation to diets with increasing protein intakes. *Clinical Science*.

[4] Boirie Y, Gachon P, Corny S, Fauquant J, Maubois JL, Beaufrère B (1996). Acute postprandial changes in leucine metabolism as assessed with an intrinsically labeled milk protein. *American Journal of Physiology*.

[5] Volpi E, Lucidi P, Cruciani G (1996). Contribution of amino acids and insulin to protein anabolism during meal absorption. *Diabetes*.

[6] Prod’homme M, Rieu I, Balage M, Dardevet D, Grizard J (2004). Insulin and amino acids both strongly participate to the regulation of protein metabolism. *Current Opinion in Clinical Nutrition and Metabolic Care*.

[7] Balage M, Averous J, Rémond D (2010). Presence of low-grade inflammation impaired postprandial stimulation of muscle protein synthesis in old rats. *Journal of Nutritional Biochemistry*.

[8] Rieu I, Sornet C, Grizard J, Dardevet D (2004). Glucocorticoid excess induces a prolonged leucine resistance on muscle protein synthesis in old rats. *Experimental Gerontology*.

[9] Rieu I, Magne H, Savary-Auzeloux I (2009). Reduction of low grade inflammation restores blunting of postprandial muscle anabolism and limits sarcopenia in old rats. *Journal of Physiology*.

[10] Lang CH, Frost RA (2006). Glucocorticoids and TNF*α* interact cooperatively to mediate sepsis-induced leucine resistance in skeletal muscle. *Molecular Medicine*.

[11] Marzani B, Balage M, Vénien A (2008). Antioxidant supplementation restores defective leucine stimulation of protein synthesis in skeletal muscle from old rats. *Journal of Nutrition*.

[12] Dardevet D, Sornet C, Bayle G, Prugnaud J, Pouyet C, Grizard J (2002). Postprandial stimulation of muscle protein synthesis in old rats can be restored by a leucine-supplemented meal. *Journal of Nutrition*.

[13] Katsanos CS, Kobayashi H, Sheffield-Moore M, Aarsland A, Wolfe RR (2006). A high proportion of leucine is required for optimal stimulation of the rate of muscle protein synthesis by essential amino acids in the elderly. *American Journal of Physiology*.

[14] Combaret L, Dardevet D, Rieu I (2005). A leucine-supplemented diet restores the defective postprandial inhibition of proteasome-dependent proteolysis in aged rat skeletal muscle. *Journal of Physiology*.

[15] Dardevet D, Sornet C, Balage M, Grizard J (2000). Stimulation of in vitro rat muscle protein synthesis by leucine decreases with age. *Journal of Nutrition*.

[16] Balage M, Dardevet D (2010). Long-term effects of leucine supplementation on body composition. *Current Opinion in Clinical Nutrition and Metabolic Care*.

[17] Pansarasa O, Flati V, Corsetti G, Brocca L, Pasini E, D’Antona G (2008). Oral amino acid supplementation counteracts age-induced sarcopenia in elderly rats. *American Journal of Cardiology*.

[18] Verhoeven S, Vanschoonbeek K, Verdijk LB (2009). Long-term leucine supplementation does not increase muscle mass or strength in healthy elderly men. *American Journal of Clinical Nutrition*.

[19] Zéanandin G, Balage M, Schneider SM, Dupont J, Mothe-Satney I (2012). Long-term leucine-enriched diet increases adipose tissue mass without affecting skeletal muscle mass and overall insulin sensitivity in old rats. *Age*.

[20] Dangin M, Boirie Y, Guillet C, Beaufrère B (2002). Influence of the protein digestion rate on protein turnover in young and elderly subjects. *Journal of Nutrition*.

[21] Rieu I, Balage M, Sornet C (2007). Increased availability of leucine with leucine-rich whey proteins improves postprandial muscle protein synthesis in aging rats. *Nutrition*.

[22] Magne H, Savary-Auzeloux I, Migné C (2012). Contrarily to whey and high protein diets, dietary free leucine supplementation cannot reverse the lack of recovery of muscle mass after prolonged immobilization during ageing. *Journal of Physiology*.

[23] Boirie Y, Dangin M, Gachon P, Vasson MP, Maubois JL, Beaufrère B (1997). Slow and fast dietary proteins differently modulate postprandial protein accretion. *Proceedings of the National Academy of Sciences of the United States of America*.

[24] Arnal MA, Mosoni L, Boirie Y (1999). Protein pulse feeding improves protein retention in elderly women. *American Journal of Clinical Nutrition*.

[25] Arnal MA, Mosoni L, Boirie Y (2000). Protein turnover modifications induced by the protein feeding pattern still persist after the end of the diets. *American Journal of Physiology*.

[26] Arnal MA, Mosoni L, Dardevet D (2002). Pulse protein feeding pattern restores stimulation of muscle protein synthesis during the feeding period in old rats. *Journal of Nutrition*.

[27] Scognamiglio R, Avogaro A, Negut C, Piccolotto R, Vigili de Kreutzenberg S, Tiengo A (2004). The effects of oral amino acid intake on ambulatory capacity in elderly subjects. *Aging*.

[28] Scognamiglio R, Piccolotto R, Negut C, Tiengo A, Avogaro A (2005). Oral amino acids in elderly subjects: effect on myocardial function and walking capacity. *Gerontology*.

[29] Scognamiglio R, Testa A, Aquilani R, Dioguardi FS, Pasini E (2008). Impairment in walking capacity and myocardial function in the elderly: is there a role for nonpharmacologic therapy with nutritional amino acid supplements?. *American Journal of Cardiology*.

[30] Solerte SB, Gazzaruso C, Bonacasa R (2008). Nutritional supplements with oral amino acid mixtures increases whole-body lean mass and insulin sensitivity in elderly subjects with sarcopenia. *American Journal of Cardiology*.

[31] Harris TB, Ferrucci L, Tracy RP (1999). Associations of elevated interleukin-6 and C-reactive protein levels with mortality in the elderly. *American Journal of Medicine*.

[32] Bautmans I, Njemini R, Lambert M, Demanet C, Mets T (2005). Circulating acute phase mediators and skeletal muscle performance in hospitalized geriatric patients. *Journals of Gerontology Series A*.

[33] Schaap LA, Pluijm SMF, Deeg DJH, Visser M (2006). Inflammatory markers and loss of muscle mass (Sarcopenia) and strength. *American Journal of Medicine*.

[34] Visser M, Pahor M, Taaffe DR (2002). Relationship of interleukin-6 and tumor necrosis factor-*α* with muscle mass and muscle strength in elderly men and women: the health ABC study. *Journals of Gerontology Series A*.

[35] Mosoni L, Balage M, Vazeille E (2010). Antioxidant supplementation had positive effects in old rat muscle, but through better oxidative status in other organs. *Nutrition*.

[36] Smith GI, Atherton P, Reeds DN (2011). Dietary omega-3 fatty acid supplementation increases the rate of muscle protein synthesis in older adults: a randomized controlled trial. *American Journal of Clinical Nutrition*.

[37] Burd NA, Wall BY, Van Loon LJC (2012). The curious case of anabolic resistance: old wives' tales or new fables?. *Journal of Applied Physiology*.

